# Depression, smoking, and lung cancer vulnerability: Bridging mental-physical comorbidity through population-based evidence

**DOI:** 10.18332/tid/207913

**Published:** 2025-09-10

**Authors:** Yibo Lu, Hui Chen, Ji Gan, Junlan Cai, Chunnuan Huang, Quanzhi Chen

**Affiliations:** 1 Medical Imaging Department, Fourth People's Hospital of Nanning, Nanning, China; 2 School of Basic Medical Sciences, Guangxi Medical University, Nanning, China

**Keywords:** NHANES, pulmonary neoplasms, depressive disorder, smoking cessation, mental health

## Abstract

**INTRODUCTION:**

The complex relationship between smoking, depression, and lung cancer remains inadequately understood, particularly regarding smoking's association with depression risk among lung cancer patients. This study examines these interactions in a nationally representative sample.

**METHODS:**

This secondary dataset analysis used cross-sectional data from 1539 US adults aged ≥20 years from the pooled 2005–2016 National Health and Nutrition Examination Survey (NHANES). We employed survey-weighted logistic regression analyses to assess associations, adjusting for sociodemographic factors and clinical comorbidities.

**RESULTS:**

Weighted prevalence estimates were 3.14% (95% CI: 2.78–3.55) for lung cancer, 29.4% for current smoking (95% CI: 28.0–30.9), and 11.18% (95% CI: 10.12–12.34) for clinically significant depression (PHQ-9 ≥10) Females had significantly higher depression prevalence than males (AOR=2.18; 95% CI: 1.63–2.91; p<0.01). Current smokers demonstrated 3.12-fold higher odds of depression compared to non-smokers (AOR=3.12, 95% CI: 2.18–4.47; p<0.001). Recent quitters (<1 year) also showed elevated depression risk (AOR=2.89; 95% CI 1.15–7.25; p=0.024). Among participants with lung cancer, current smokers had a significantly higher prevalence of depression compared to non-smokers (16.82% vs 4.12%; p=0.0008).

**CONCLUSIONS:**

Smoking was strongly associated with depression in lung cancer patients, with recent cessation representing a high-risk period. Integrated smoking cessation and mental health interventions are needed, particularly for young females.

## INTRODUCTION

The American Cancer Society makes estimates of the number of new cancer cases and deaths every year. Cancer mortality continued to decline through 2021, with more than 4 million deaths avoided since 1991 due to reductions in smoking, earlier detection of certain cancers, and improvements in adjuvant and metastatic treatment regimens in the United States^1^. Lung cancer is one of the most commonly diagnosed cancers globally and is the leading cause of cancer-related deaths worldwide, with an estimated 2 million new cases and 1.76 million deaths annually^2^. Lung cancer has been identified as one of the cancers most significantly influenced by smoking, which is a major risk factor for cancer development and closely related to its diagnosis and treatment^3^.

Nowadays, increasing attention has been paid to the mental health status of lung cancer patients, particularly the prevalence of depression, which is closely related to the treatment of lung cancer patients^4^. Addressing the depression in lung cancer patients has positive implications for improving their mental health and treatment, as well as alleviating the distress during survival^5^. Additionally, smoking, a significant factor related to lung cancer, is also closely associated with depression, and genetic susceptibility to depression could increase the likelihood of smoking^6^.

Emerging evidence suggests that smoking may act as a bidirectional mediator between depression and lung carcinogenesis^7^. On the one hand, nicotine dependence could exacerbate depressive symptoms through neuroinflammatory pathways^8^. On the other hand, depression-related cognitive impairments may hinder smoking cessation efforts^9^. However, three critical gaps persist. First, most studies focus on pairwise associations (smoking-cancer or smoking-depression), neglecting their synergistic effects. Second, the moderating role of demographic factors remains underexplored. Third, population-based evidence integrating biochemical markers is scarce.

The National Health and Nutrition Examination Survey (NHANES) provides a unique opportunity to investigate these relationships in a nationally representative population^10^. Building on prior evidence of smoking-depression comorbidity, we hypothesize that quitters will exhibit higher depression prevalence than never smokers, reflecting withdrawal-related mood dysregulation and that smoking status mediates the depression-lung cancer association through both biological and behavioral pathways. This study aims to test these hypotheses by characterizing population-level patterns and quantifying mediation effects, leveraging NHANES’s strengths: nationally representative sampling, standardized mental health assessments, and biochemical validation of smoking status.

## METHODS

### Data source

This secondary dataset analysis utilized pooled data from the National Health and Nutrition Examination Survey (NHANES) 2005–2016, a nationally representative cross-sectional survey conducted by the National Center for Health Statistics (NCHS). NHANES employs a complex, multistage probability sampling design to ensure generalizability to the non-institutionalized US population. The survey integrates interviews, physical examinations, and biological specimen collection, with ethical oversight provided by the NCHS Research Ethics Review Board (Protocol #2005–06 and subsequent continuations).

### Data processing

**Figure 1:**
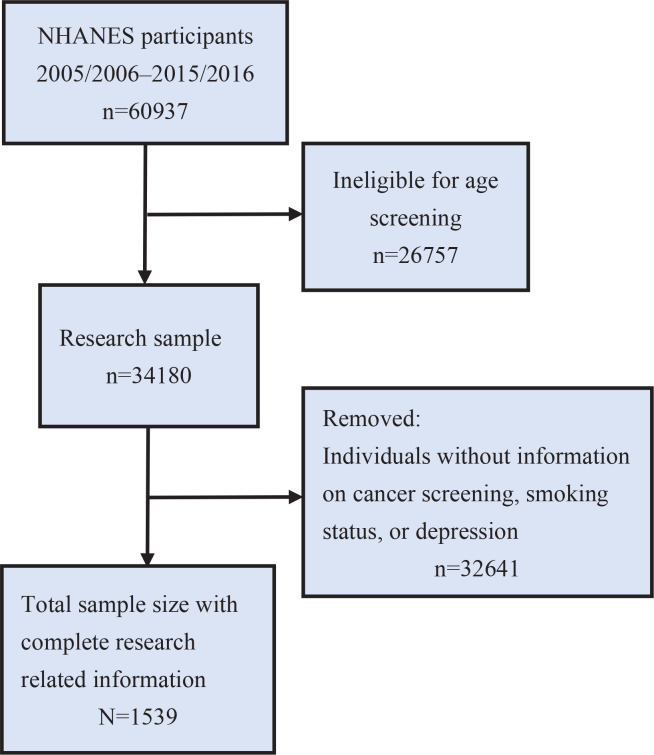
Flowchart of inclusion of participants aged ≥20 years in the cross-sectional analysis of depression and lung cancer mediated by smoking status, National Health and Nutrition Examination Survey (NHANES), United States, 2005–2016 (N=1539)

Data processing and analysis were performed using R software with the *dplyr* and *survey* packages. We obtained demographic, lung cancer, smoking status, and depression screening (PHQ-9) data for the 2005–2016 cycles from the NHANES database. Data from different years were merged to create a unified dataset. To account for the complex survey design of NHANES, we created a weighted analysis object incorporating sampling weights, stratification variables, and primary sampling units. Participants were screened according to the following eligibility criteria: age ≥20 years; complete data on depression status (assessed by the Patient Health Questionnaire-9, PHQ-9); documented cancer diagnosis history (self-reported or medically confirmed); and exclusion of pregnant women and individuals with incomplete demographic/clinical covariates. Participants were screened following the steps in Figure 1.

### Variable definitions

Smoking status was categorized as: 1) Non-smokers – not currently smoking (SMQ040 = 3); and 2) Current smokers – daily/occasional cigarette smokers (SMQ040 =1 or 2). Smoking cessation status was categorized as: 1) Recent quitters – patients that had already quit smoking (SMQ050 = 1–66); and 2) Other –smoking cessation status of these patients was unknown. (SMQ050 = 77777 or 9999). Depression was defined^8^ by PHQ-9 scores ≥10.

### Statistical analysis

All analyses accounted for NHANES’s complex survey design using R’s survey package (v4.1) with the WTINT2YR sampling weights. After confirming data were missing completely at random (tested via Little’s test), we performed multiple imputation^11^ using chained equations for incomplete covariates, generating 20 imputed datasets. A total of 1539 adults were included in this dataset after applying strict exclusion criteria to the original 60937 respondents, with removal of participants missing critical variables for our analyses, which included lung cancer diagnosis (n=892), smoking status (n=1243), and PHQ-9 depression scores (n=57263). The resulting sample maintained demographic representativeness through NHANES survey weighting, with non-response adjustments applied to preserve national population estimates.

Weighted prevalence estimates with 95% confidence intervals (CIs) were calculated using the Korn-Graubard-adjusted *svyciprop()* function. Multivariable logistic regression via *svyglm()* adjusted for age (20–39, 40–59, ≥60 years), sex, race, education level, income-to-poverty ratio, and comorbidities. Variance was estimated using Taylor linearization.

Effect modification was assessed through Wald tests of interaction terms. Sensitivity analyses included complete-case comparisons and multicollinearity diagnostics (VIFs <2.5).

## RESULTS

### Study population

The final analytic study population comprised 1539 adults (55.7% male, 44.3% female; age range 20–85 years, with a mean age of 65.4 ± 24.1 years) selected from NHANES 2005–2016 (Table 1).

**Table 1:** Study population characteristics, NHANES 2005–2016 (N=1539)

*Characteristics*	*n*
Total participants	60937
Age ≥20 years	34180
Invalid cases	32641
Effective number of cases	1539
Males	857
Females	682
Age range (years)	20–85

### Lung cancer prevalence

Weighted analyses revealed a 3.14% (95% CI: 2.78–3.55) overall prevalence of self-reported lung cancer diagnosis. Subgroup evaluations showed gender disparities as males exhibited a 3.97% prevalence (OR=0.62; 95% CI: 0.34–1.13; AOR=0.58; 95% CI 0.30–1.12; p=0.114). The attenuation of the odds ratio after adjustment suggests potential confounding by age or comorbidities. Multivariable models adjusted for age, sex, race/ethnicity, income-to-poverty ratio, and comorbidities (COPD, cardiovascular disease) to control for socioeconomic and health status confounders. Current smokers showed marginally lower prevalence (AOR=1.21; 95% CI: 0.67–2.18; p=0.515). Full stratified estimates are available in Table 2.

**Table 2:** Distribution of lung cancer prevalence with adjusted odds ratios, NHANES 2005–2016 (N=1539)

*Variables*	*Weighted %* *(95% CI)*	*p*	*OR (95% CI)*	*AOR (95% CI)*
**Total**	3.14 (2.78–3.55)			
**Sex**		0.114		
Male ^®^	3.97 (2.45–5.49)		1	1
Female	2.35 (1.53–3.17)		0.62 (0.34–1.13)	0.58 (0.30–1.12)
**Age** (years)		0.174		
20–39 ^®^	2.14 (0.00–4.66)		1	1
40–59	2.01 (0.29–3.73)		0.94 (0.31–2.85)	0.94 (0.31–2.85)
≥60	3.80 (2.51–5.09)		1.82 (0.67–4.95)	1.82 (0.67–4.95)
**Smoking status**		0.515		
Current smokers	2.64 (1.82–3.46)		0.78 (0.52–1.17)	1.21 (0.67–2.18)
Non-smokers ^®^	3.35 (2.71–3.99)		1	1
**Smoking cessation**		0.305		
Recent quitters	8.96 (4.62–13.30)		2.34 (0.81–6.75)	2.34 (0.81–6.75)
Other ^®^	3.08 (2.58–3.58)		1	1
**Depression** (PHQ-9 ≥10)		0.167		
Yes	2.04 (1.02–3.06)		0.72 (0.45–1.15)	0.72 (0.45–1.15)
No^®^	3.28 (2.84–3.72)		1	1

AOR: adjusted odds ratio. Multivariable models adjusted for age, sex, race/ethnicity, income-to-poverty ratio, and comorbidities (COPD, cardiovascular disease). Crude ORs derived from univariate logistic regression. Smoking status was categorized as: 1) Non-smokers – not currently smoking (SMQ040 = 3), 2) Current smokers – daily/occasional cigarette smokers (SMQ040 = 1 or 2). Smoking cessation status was categorized as: 1) Recent quitters – patients that had already quit smoking (SMQ050 = 1–66), 2) Other – smoking cessation status of these patients was unknown (SMQ050 = 77777 or 9999). ^®^ Reference categories.

### Current smoking patterns

Current smoking prevalence reached 29.41% (95% CI: 28.02–30.85) in our subsample, with significant demographic differentials as females demonstrated higher odds of current smoking (OR=1.62; 95% CI: 1.34–1.96; AOR=1.97; 95% CI: 1.42–2.73; p<0.05) compared to males. This association persisted after adjusting for age, race/ethnicity, and income, compared to males, contradicting historical sex patterns in tobacco use. Smoking prevalence was inversely correlated with age, peaking at 69.37% (95% CI: 65.12–73.62) in those aged 20–39 years (AOR=1.00, Ref.) and declining to 16.99% (95% CI: 14.67–19.31) in those ≥60 years (AOR=0.12; 95% CI: 0.08–0.17; p<0.001). With regard to mental health associations, individuals with a PHQ-9 ≥10 exhibited higher smoking rates (56.30%; 95% CI: 51.23–61.37) compared to asymptomatic individuals (AOR=3.82; 95% CI: 2.65–5.51; p<0.001) when adjusting for age, sex, and income. Recent quitters constituted only 1.02% (95% CI: 0.65–1.40) of the population, with no significant subgroup variations. This contrasts with the 8.96% prevalence of recent quitters in Table 2, suggesting high relapse rates in this population. Detailed smoking behavior profiles are shown in Table 3.

**Table 3:** Weighted prevalence and adjusted odds ratios for smoking status, NHANES 2005–2016 (N=1539)

*Variables*	*Current smokers* *% (95% CI)*	*AOR (95% CI)*	*Recent quitters* *% (95% CI)*	*AOR (95% CI)*
**Total**	29.41 (28.02–30.85)		1.02 (0.65–1.40)	
**Sex**				
Male ^®^	22.46 (18.23–26.68)	1	1.06 (0.27–1.85)	1
Female	36.12 (30.79–41.46)	1.97 (1.42–2.73)*	0.98 (0.16–1.81)	0.92 (0.33–2.56)
**Age** (years)				
20–39 ^®^	69.37 (65.12–73.62)	1	1.85 (0.00–3.92)	1
40–59	45.76 (41.32–50.20)	0.38 (0.28–0.52)*	1.76 (0.85–2.67)	0.95 (0.31–2.92)
≥60	16.99 (14.67–19.31)	0.12 (0.08–0.17)*	0.57 (0.20–0.94)	0.31 (0.09–1.07)
**Lung cancer**				
Yes	24.74 (18.23–31.25)	0.78 (0.45–1.35)	2.91 (1.23–4.59)	0.31 (0.09–1.07)
No ^®^	29.56 (28.12–31.00)	1	0.96 (0.58–1.34)	1
**Depression** (PHQ-9 ≥10)				
Yes	56.30 (51.23–61.37)	3.82 (2.65–5.51)*	2.52 (1.12–3.92)	2.43 (1.05–5.62)*
No ^®^	26.03 (24.52–27.54)	1	0.83 (0.51–1.15)	1

AOR: adjusted odds ratio. Multivariable models adjusted for age, sex, race/ethnicity, income-to-poverty ratio, and depression status. Smoking status was categorized as: 1) Non-smokers – not currently smoking (SMQ040 = 3), 2) Current smokers – daily/occasional cigarette smokers (SMQ040 = 1 or 2). Smoking cessation status was categorized as: 1) Recent quitters – patients that had already quit smoking (SMQ050 = 1–66), 2) Other – smoking cessation status of these patients was unknown (SMQ050 = 77777 or 9999). Lung cancer diagnosis includes self-report and medical records.

* p<0.05.

^®^ Reference categories.

### Depression burden

The weighted prevalence of clinically significant depression (PHQ-9 ≥10)^10^ reached 11.18% (95% CI: 10.12–12.34). Notable disparities emerged across demographic and behavioral strata. Females exhibited nearly double the depression prevalence of males (OR=2.12; 95% CI: 1.43–3.14; AOR=2.18; 95% CI: 1.63–2.91; p<0.01). This elevated risk persisted after adjusting for sociodemographic and socioeconomic factors. Depression prevalence demonstrated a marked inverse association with age, declining from 25.16% (95% CI: 21.38–28.94) in young adults (18–34 years) to 6.69% (95% CI: 5.42–7.96) among seniors (≥65 years). Compared to young adults, seniors showed 76% reduced odds of depression (AOR=0.24; 95% CI: 0.17–0.34; p<0.001). Compared to non-smokers, current smokers demonstrated tripled depression risk (AOR=3.12; 95% CI: 2.18–4.47; p<0.001). Recent quitters also showed elevated risk (AOR=2.89; 95% CI: 1.15–7.25; p=0.024), though with wider confidence intervals reflecting smaller subgroup size. Comprehensive metrics for all subgroups are presented in Table 4.

**Table 4:** Weighted prevalence and adjusted odds ratios for depression, by demographic and behavioral characteristics, NHANES 2005–2016 (N=1539)

*Variables*	*Crude % (95% CI)*	*Adjusted % (95% CI)†*	*AOR (95% CI)*
**Total**	11.18 (10.12–12.34)	10.95 (9.88–12.02)	
**Sex**			
Male ^®^	7.34 (5.09–9.59)	6.89 (4.72–9.06)	1
Female	14.88 (12.47–17.29)	14.12 (11.83–16.41)	2.18 (1.63–2.91)**
**Age** (years)			
20–39 ^®^	25.16 (21.38–28.94)	23.87 (20.25–27.49)	1
40–59	17.19 (14.21–20.17)	16.54 (13.72–19.36)	0.62 (0.45–0.85)**
≥60	6.69 (5.42–7.96)	6.32 (5.11–7.53)	0.24 (0.17–0.34)***
**Lung cancer**			
Yes	7.26 (4.31–10.21)	6.95 (4.12–9.78)	0.82 (0.52–1.29)
No ^®^	11.30 (10.20–12.40)	11.02 (9.94–12.10)	1
**Smoking status**			
Current smokers	21.39 (18.15–24.64)	20.01 (17.02–23.00)	3.12 (2.18–4.47)***
Non-smokers ^®^	6.91 (5.82–8.00)	6.32 (5.20–7.44)	1
**Smoking cessation**			
Recent quitters	27.58 (13.84–41.32)	25.94 (12.95–38.93)	2.89 (1.15–7.25)*
Other ^®^	11.01 (9.90–12.12)	10.78 (9.70–11.86)	1

AOR: adjusted odds ratio.

† Adjusted for age, sex, race/ethnicity, income-to-poverty ratio, and education level. All estimates incorporate NHANES survey weights (WTINT2YR). Smoking status was categorized as: 1) Non-smokers – not currently smoking (SMQ040 = 3), 2) Current smokers – daily/occasional cigarette smokers (SMQ040 = 1 or 2). Smoking cessation status was categorized as: 1) Recent quitters – patients that had already quit smoking (SMQ050 = 1–66), 2) Other – smoking cessation status of these patients was unknown (SMQ050 = 77777 or 9999). Lung cancer diagnosis includes self-report and medical records.

* p<0.05,

** p<0.01,

*** p<0.001.

^®^ Reference categories.

Sensitivity analyses confirmed the robustness of these findings: Complete-case versus imputed models showed minimal parameter divergence (ΔAOR<10% for smoking status effects). All variance inflation factors (VIFs) remained <2.5, indicating absence of multicollinearity.

## DISCUSSION

The global burden of lung cancer reflects heterogeneous risk factor landscapes, with smoking prevalence, environmental exposures, and socioeconomic conditions driving regional disparities in incidence and mortality^12^. While lung cancer remains the leading cause of cancer-related death worldwide^1^, our analysis of NHANES 2005–2016 data provides critical insights into the US adult population, demonstrating persistent challenges in smoking control and mental health comorbidity management.

This NHANES analysis reveals three central patterns. Depression was strongly associated with smoking behavior, with current smokers and recent quitters showing 3.12-fold and 2.89-fold higher odds of depression compared to non-smokers. Gender modifies this relationship, as female smokers exhibit 44% greater depression vulnerability than males. Smoking status showed no significant association with lung cancer prevalence in this cohort. However, among the subset with lung cancer, smokers exhibited significantly higher depression burden, while the non-significant statistical association in our study population may reflect methodological limitations inherent to cross-sectional survey designs.

The current smoking prevalence in our study population was 29.4%, while the minimal representation of recent quitters (1.02%) reflects persistent challenges in smoking cessation, particularly among individuals with comorbid mental illness^13^. Our finding that 56.3% of current smokers exhibit clinically significant depression (PHQ-9 ≥10) aligns with longitudinal evidence demonstrating smoking’s role in both depression etiology and maintenance^14,15^. Notably, recent quitters exhibit even higher adjusted depression prevalence (25.94%; 95% CI: 12.95–38.93) compared to current smokers (20.01%; 95% CI: 17.02–23.00), with 2.89-fold elevated odds (95% CI: 1.15–7.25) after controlling for sociodemographic factors. This paradoxical association underscores nicotine’s self-medication properties in acute depression relief^16^, while the residual neurobiological effects of smoking cessation may increase vulnerability to depressive relapse^17^. Moreover, a shared genetic risk architecture between nicotine dependence and major depressive disorder has also been noted^18^. The strong association between smoking and depression in our analyses may reflect complex temporal relationships, though cross-sectional data preclude causal inference, creating a maintenance cycle difficult to disrupt through cessation alone.

Our exploratory analysis in lung cancer patients suggested current smokers had higher depression prevalence than non-smokers (16.82% vs 4.12%; p=0.0008). However, small sample size and potential residual confounding require cautious interpretation. This association persisted after adjusting for sociodemographic confounders, suggesting behavioral or biological pathways warranting further investigation. Notably, independent analyses showed females had a 2.18-fold higher likelihood of depression than males, and young adults (20–39 years) had the highest depression prevalence (25.16%). This suggests young female smokers may represent a vulnerable subgroup, though direct interaction testing was not performed. The 4.1-fold odds of depression among smoking patients may reflect the direct neurotoxic effects of smoking on serotonergic pathways^6^, psychosocial stressors from stigma and social isolation, or reversed causation where pre-existing depression impedes cessation efforts^19^.

Our findings reinforce the need for integrated oncology-psychiatry care models. While smoking cessation remains the cornerstone of lung cancer prevention, attention to mental health sequelae is needed as we identified 2.89-fold elevated odds of depression in recent quitters. Moreover, longitudinal evidence links depression to higher mortality in early-stage disease^20^, and sex disparities in treatment adherence^21^. The 25.2% depression prevalence among young adults in our sample (20–39 years) may imply that screen-and-treat protocols should extend beyond traditional oncology settings.

### Limitations

Our study has limitations that warrant consideration. The cross-sectional design precludes causal inference regarding the smoking-depression-lung cancer triad. Self-reported lung cancer diagnoses may underestimate true prevalence due to survival bias and lack of histological confirmation. Residual confounding from unmeasured variables – including secondhand smoke exposure, alcohol use, physical activity, body mass index (BMI), urbanization, and genetic susceptibility – persists despite multivariable adjustment as these factors are established depression risk predictors^22-24^ and may confound the observed associations. The US-specific healthcare context may not reflect settings with different healthcare systems, and NHANES sampling excludes institutionalized populations, potentially underestimating severe comorbidities. Absence of formal mediation analysis limits causal interpretation of smoking’s mediating role.

Despite these limitations, the nationally representative data and structured mental health assessments provide robust population-based evidence. Future research should conduct multinational cohort studies to examine cultural modifiers, implement causal mediation analyses with time-varying exposures and potentially integrate multi-omics data (e.g. epigenetics) to elucidate biological pathways. Moreover, future studies should adjust for physical activity, BMI, and urbanization in depression-smoking models.

## CONCLUSIONS

Despite methodological constraints, this study indicates that recent smoking cessation (<1 year) is associated with higher odds of depression. Current smokers have greater odds of depression compared to non-smokers. Among lung cancer patients, smoking shows a preliminary link to depression burden. Integrated interventions should focus on depression management during cessation, especially for females and young adults.

## Data Availability

The data supporting this research are available from the authors on reasonable request.

## References

[1] Siegel RL, Giaquinto AN, Jemal A. Cancer statistics, 2024. CA Cancer J Clin. 2024;74(1):12-49. doi:10.3322/caac.21820 38230766

[2] Thai AA, Solomon BJ, Sequist LV, Gainor JF, Heist RS. Lung cancer. Lancet. 2021;398(10299):535-554. doi:10.1016/S0140-6736(21)00312-3 34273294

[3] Dizon DS, Kamal AH. Lung cancer screening guidelines: smoking matters, not quitting. CA Cancer J Clin. 2024;74(1):10-11. doi:10.3322/caac.21814 37909864

[4] Zeng Y, Hu CH, Li YZ, et al. Association between pretreatment emotional distress and immune checkpoint inhibitor response in non-small-cell lung cancer. Nat Med. 2024;30(6):1680-1688. doi:10.1038/s41591-024-02929-4 38740994 PMC11186781

[5] Xiao J, Chow KM, Choi KC, et al. Effects of family-oriented dignity therapy on dignity, depression and spiritual well-being of patients with lung cancer undergoing chemotherapy: A randomised controlled trial. Int J Nurs Stud. 2022;129:104217. doi:10.1016/j.ijnurstu.2022.104217 35339908

[6] Yao Y, Xu Y, Cai Z, et al. Determination of shared genetic etiology and possible causal relations between tobacco smoking and depression. Psychol Med. 2021;51(11):1870-1879. doi:10.1017/S003329172000063X 32249730

[7] Zhu X, Ye R, Jiang X, Zhang J. Smoking as a mediator in the association between major depressive disorder and schizophrenia on lung cancer risk: a bidirectional/multivariable and mediation Mendelian randomization study. Front Psychiatry. 2024;15:1367858. doi:10.3389/fpsyt.2024.1367858 39176232 PMC11338888

[8] Kim T, Han J, Kwon S. Nicotine dependence comorbid with depressive symptoms may limit immediate cognitive improvement following exercise. Physiol Behav. 2025;291:114809. doi:10.1016/j.physbeh.2025.114809 39793846

[9] Rae J, Pettey D, Aubry T, Stol J. Factors affecting smoking cessation efforts of people with severe mental illness: a qualitative study. J Dual Diagn. 2015;11(1):42-49. doi:10.1080/15504263.2014.992096 25491704

[10] Kroenke K, Spitzer RL, Williams JB. The PHQ-9: validity of a brief depression severity measure. J Gen Intern Med. 2001;16(9):606-613. doi:10.1046/j.1525-1497.2001.016009606.x 11556941 PMC1495268

[11] Spitzer RL, Kroenke K, Williams JB. Validation and utility of a self-report version of PRIME-MD: the PHQ primary care study. Primary care evaluation of mental disorders. Patient Health Questionnaire. JAMA. 1999;282(18):1737-1744. doi:10.1001/jama.282.18.1737 10568646

[12] Zhu H, Fu Q, Chen R, Luo L, Yu M, Zhou Y. Association of dietary decanoic acid intake with diabetes or prediabetes: an analysis from NHANES 2005-2016. Front Nutr. 2025;11:1483045. doi:10.3389/fnut.2024.1483045. 39839274 PMC11747714

[13] Blazek K, van Zwieten A, Saglimbene V, Teixeira-Pinto A. A practical guide to multiple imputation of missing data in nephrology. Kidney Int. 2021;99(1):68-74. doi:10.1016/j.kint.2020.07.035 32822702

[14] Glassman AH, Helzer JE, Covey LS, et al. Smoking, smoking cessation, and major depression. JAMA. 1990;264(12):1546-1549. 2395194

[15] Boden JM, Fergusson DM, Horwood LJ. Cigarette smoking and depression: tests of causal linkages using a longitudinal birth cohort. Br J Psychiatry. 2010;196(6):440-446. doi:10.1192/bjp.bp.109.065912 20513853

[16] Khaled SM, Bulloch AG, Williams JV, Hill JC, Lavorato DH, Patten SB. Persistent heavy smoking as risk factor for major depression (MD) incidence - Εvidence from a longitudinal Canadian cohort of the national population health survey. J Psychiatr Res. 2012;46(4):436-443. doi:10.1016/j.jpsychires.2011.11.011 22277304

[17] Munafò MR, Araya R. Cigarette smoking and depression: a question of causation. Br J Psychiatry. 2010;196(6):425-426. doi:10.1192/bjp.bp.109.074880 20513848

[18] Balfour DJ, Ridley DL. The effects of nicotine on neural pathways implicated in depression: a factor in nicotine addiction? Pharmacol Biochem Behav. 2000;66(1):79-85. doi:10.1016/s0091-3057(00)00205-7 10837846

[19] Thorndike AN, Regan S, McKool K, et al. Depressive symptoms and smoking cessation after hospitalization for cardiovascular disease. Arch Intern Med. 2008;168(2):186-191. doi:10.1001/archinternmed.2007.60 18227366

[20] Sullivan DR, Forsberg CW, Ganzini L, et al. Longitudinal changes in depression symptoms and survival among patients with lung cancer: a national cohort assessment. J Clin Oncol. 2016;34(33):3984-3991. doi:10.1200/JCO.2016.66.8459 27996350 PMC5477833

[21] Morrison EJ, Novotny PJ, Sloan JA, et al. Emotional problems, quality of life, and symptom burden in patients with lung cancer. Clin Lung Cancer. 2017;18(5):497-503. doi:10.1016/j.cllc.2017.02.008 28412094 PMC9062944

[22] Schuch FB, Vancampfort D, Firth J, et al. Physical activity and incident depression: a meta-analysis of prospective cohort studies. Am J Psychiatry. 2018;175(7):631-648. doi:10.1176/appi.ajp.2018.17111194 29690792

[23] Stunkard AJ, Faith MS, Allison KC. Depression and obesity. Biol Psychiatry. 2003;54(3):330-337. doi:10.1016/s0006-3223(03)00608-5 12893108

[24] Purtle J, Nelson KL, Yang Y, Langellier B, Stankov I, Diez Roux AV. Urban-rural differences in older adult depression: a systematic review and meta-analysis of comparative studies. Am J Prev Med. 2019;56(4):603-613. doi:10.1016/j.amepre.2018.11.008 30777704

